# The Long-Acting Serine Protease Inhibitor mPEG-SPA-MDSPI16 Alleviates LPS-Induced Acute Lung Injury

**DOI:** 10.3390/ijms25084567

**Published:** 2024-04-22

**Authors:** Jingrui Chen, Xinjun Zhou, Nan Dai, Xiaoyu Liu, Shihan Liu, Haipeng Zhang, Lingcong Kong, Hongxia Ma

**Affiliations:** 1 College of Veterinary Medicine, Jilin Agricultural University, Xincheng Street No. 2888, Changchun 130118, China; chenjingrui@jlau.edu.cn; 2 The Key Laboratory of New Veterinary Drug Research and Development of Jilin Province, Jilin Agricultural University, Xincheng Street No. 2888, Changchun 130118, China; 3 College of Life Science, Jilin Agricultural University, Xincheng Street No. 2888, Changchun 130118, China; k_sz0803@163.com (X.Z.); 20220828@mails.jlau.edu.cn (N.D.); liushihan@mails.jlau.edu.cn (S.L.); zhp@jlau.edu.cn (H.Z.); 4 The Engineering Research Center of Bioreactor and Drug Development, Ministry of Education, Jilin Agricultural University, Xincheng Street No. 2888, Changchun 130118, China

**Keywords:** mPEG-SPA-MDSPI16, pharmaceutical properties, ALI, anti-inflammatory activity, anti-inflammatory mechanism

## Abstract

Anti-inflammatory drugs have become the second-largest class of common drugs after anti-infective drugs in animal clinical care worldwide and are often combined with other drugs to treat fever and viral diseases caused by various factors. In our previous study, a novel serine protease inhibitor-encoding gene (*MDSPI16*) with improved anti-inflammatory activity was selected from a constructed suppressive subducted hybridization library of housefly larvae. This protein could easily induce an immune response in animals and had a short half-life, which limited its wide application in the clinic. Thus, in this study, mPEG-succinimidyl propionate (mPEG-SPA, Mw = 5 kDa) was used to molecularly modify the MDSPI16 protein, and the modified product mPEG-SPA-MDSPI16, which strongly inhibited elastase production, was purified. It had good stability and safety, low immunogenicity, and a long half-life, and the IC_50_ for elastase was 86 nM. mPEG-SPA-MDSPI16 effectively inhibited the expression of neutrophil elastase and decreased ROS levels. Moreover, mPEG-SPA-MDSPI16 exerted anti-inflammatory effects by inhibiting activation of the NF-κB signaling pathway and the MAPK signaling pathway in neutrophils. It also exerted therapeutic effects on a lipopolysaccharide (LPS)-induced acute lung injury (ALI) mouse model. In summary, mPEG-SPA-MDSPI16 is a novel anti-inflammatory protein modified with PEG that has the advantages of safety, nontoxicity, improved stability, and strong anti-inflammatory activity in vivo and in vitro and is expected to become an effective anti-inflammatory drug.

## 1. Introduction

Acute lung injury (ALI), especially the severe form, acute respiratory distress syndrome (ARDS), is an inflammatory lung disease characterized by pulmonary edema, endothelial and epithelial damage, and neutrophil infiltration [1]. As the first line of defense in the body, neutrophils are the first cells to reach the site of inflammation and release a variety of inflammatory factors (tumor necrosis factor (TNF)-α, interleukin (IL)-1β and IL-6, etc.) and a variety of proteases involved in the inflammatory response [2]. However, an imbalance between proinflammatory and anti-inflammatory factors released by neutrophils can lead to severe morbidity and mortality. A large number of clinical trials and research data show that one of the important pathogenic mechanisms of pulmonary inflammation is the imbalance between the large amount of elastase released by overactivated neutrophils and protease inhibition [3]. Therefore, several new anti-inflammatory drugs, such as elastase inhibitors for protease hydrolysis, have been developed. Elastase inhibitors can inhibit the activity of elastase and are expected to become new agents for the treatment of pulmonary inflammation caused by excessive neutrophil activation.

Serine protease inhibitors are a kind of protein superfamily that can inhibit serine protease. These inhibitors widely exist in various viruses, bacteria, animals, and plants and participate in various immune reactions, such as inflammatory response, antiviral infection, antitumor activity, fibrinolysis, blood coagulation, and immune regulation [4]. Serine protease inhibitors have become key targets for research on immune regulation and blood coagulation functions [5]. As the only marketed elastase inhibitor drug, Sivelestat has played an important role in the treatment of pneumonia. With the continuous use of Sivelestat in the clinic, adverse reactions, such as abnormal liver function and elevated phospholipase, have also been observed [6]. Therefore, more elastase inhibitors with better anti-inflammatory effects, less toxicity, fewer side effects, and wider application ranges are needed. In a previous study, we screened a housefly inhibitory screening library and identified the serine protease inhibitor MDSPI16, which robustly inhibited elastase (IC_50_ = 13 nM, Ki = 2.8 nM) and chymotrypsin (IC_50_ = 60 nM, Ki = 28 nM), and the inhibitory effect of MDSPI16 on elastase was greater than that of carbaryl (IC_50_ = 1.7 μM, Ki = 46 nM). MDSPI16 also exerted a significant anti-inflammatory effect on lipopolysaccharide (LPS)-induced acute lung injury in mice [7]. However, this factor has not been approved for clinical use thus far due to its large molecular weight and strong immunogenicity.

With the development of biotechnology, protein molecular modification technology has been used to improve the physiological properties of proteins and can be used to solve or alleviate many problems associated with protein use. Among these methods, PEGylation is a well-known strategy for enhancing the stability of protein drugs against enzymatic degradation and can increase temperature and pH stability. PEG-conjugated proteins also have the advantages of low immunogenicity, a prolonged plasma half-life, and the avoidance of renal clearance [8]. Notably, PEGylation has been widely used since it was first approved by the FDA in 1990, and 19 PEGylated drugs are currently on the market [9,10]. These drugs include PEGylated interferon alfa-2b (PEG-Intron^®^) [11], PEGylated asparaginase (Oncaspar^®^) [12], and PEGylated adenosine deaminase (Adagen^®^) [13]. However, it is worth noting that the modification of serine protease inhibitors by PEG has never been reported, and the anti-inflammatory effects and mechanisms of the modified products at the cellular level and in animals have not been described in the literature. Therefore, in this study, MDSPI16 was modified with mPEG-succinimidyl propionate (mPEG-SPA; Mw = 5 kDa) to improve the bioavailability of the novel serine protease inhibitor MDSPI16 in vivo, and the modified product mPEG-SPA-MDSPI16 was purified. Additionally, its pharmacological properties, anti-inflammatory mechanism, and therapeutic effect on mice with LPS-induced ALI were examined. This study revealed for the first time that mPEG-SPA-MDSPI16 can ameliorate acute lung injury in mice. Our study provides a foundation for the development of a safe and effective long-acting anti-inflammatory protein.

## 2. Results

### 2.1. Preparation and Purification of mPEG-SPA-MDSPI16

**Figure 1:**
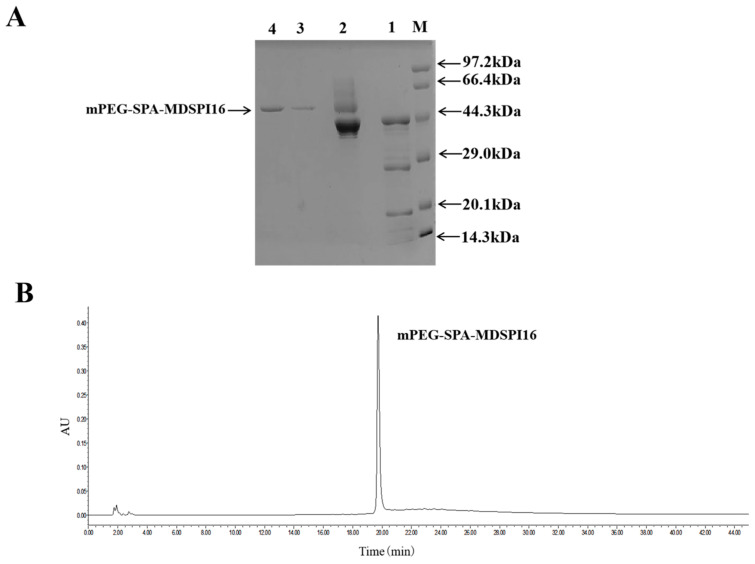
The results of preparation and purification of mPEG-SPA-MDSPI16. (**A**) mPEG-SPA-MDSPI16 was purified by SP Sepharose FF. Lane M, molecular weight standards; lane 1, purified product of MDSPI16; lane 2, PEGylation mixture; lanes 3–4, purified mPEG-SPA-MDSPI16. (**B**) Analysis of mPEG-SPA-MDSPI16 by HPLC.

SP-Sepharose FF was used to purify the modified product mPEG-SPA-MDSPI16, and the elution peaks at 280 nm were collected. The SDS-PAGE result showed only one clear band of the purified product. Compared with the band of the unpurified modified product, this band was a unit point-modified product with a molecular weight greater than that of MDSPI16 (45 kDa), and the casein plate results showed that the protein exhibited hydrolytic activity against neutrophil elastase (Figure 1A). The modified products were loaded onto a volume exclusion column using RP-HPLC; only one peak was observed when the absorbance was measured at 280 nm using a UV detector, and this peak protein could hydrolyze neutrophil elastase (Figure 1B and Figure S1).

### 2.2. Temperature and pH Stabilities of mPEG-SPA-MDSPI16

The results showed that MDSPI16 and mPEG-SPA-MDSPI16 exhibited similar inhibitory activities at 37–100 °C, and the inhibitory activity was lost after 180 min at 100 °C (Figure S2A,B). Moreover, the inhibitory activities of mPEG-SPA-MDSPI16 and MDSPI16 decreased after storage at 4 °C, and the inhibitory activity of mPEG-SPA-MDSPI16 was greater than that of MDSPI16 until the inhibitory activity of MDSPI16 completely disappeared at 70 days. However, mPEG-SPA-MDSPI16 still had 30% inhibitory activity (Figure S2C). In addition, we evaluated the effect of pH on the inhibitory activity of mPEG-SPA-MDSPI16. The pH curve of mPEG-SPA-MDSPI16 showed peak activity at pH 2.0. The inhibitory activity of mPEG-SPA-MDSPI16 was stable at pH 2-pH 11 and less than 20% at pH 13 (Figure S2D).

### 2.3. IC_50_ Analysis

The IC_50_ results showed that mPEG-SPA-MDSPI16 strongly inhibited elastase activity (IC_50_ = 86 nM). Compared with the inhibitory effect of MDSPI16 on elastase (IC_50_ = 13 nM), the inhibitory effect of mPEG-SPA-MDSPI16 was slightly decreased.

### 2.4. Safety Analysis

**Figure 2:**
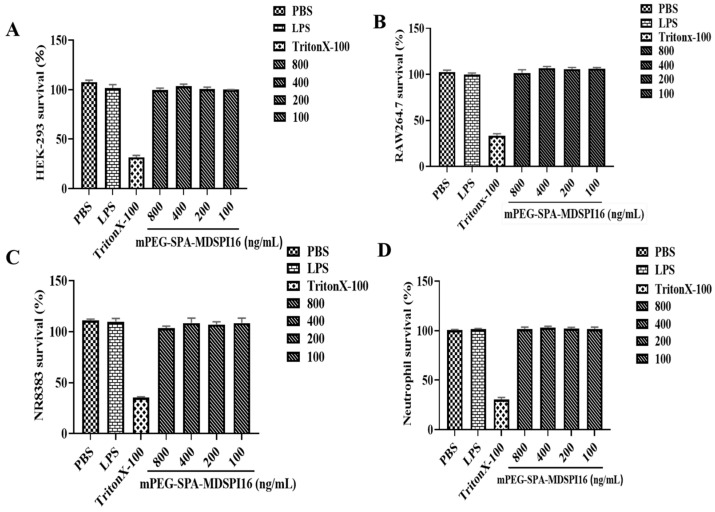
Safety evaluation of mPEG-SPA-MDSPI16 at the cell level. (**A**) Cytotoxicity of mPEG-SPA-MDSPI16 against HEK-293 cells. (**B**) Cytotoxicity of mPEG-SPA-MDSPI16 against RAW 264.7 cells. (**C**) Cytotoxicity of mPEG-SPA-MDSPI16 against NR8383 cells. (**D**) Cytotoxicity of mPEG-SPA-MDSPI16 against neutrophils. Graphs show the mean of three biological replicates; *p*-values were determined using an unpaired two-tailed Student’s *t*-test.

The hemolytic activity of mPEG-SPA-MDSPI16 is shown in Figure S3A. As the concentrations of the elastase inhibitors MDSPI16 and mPEG-SPA-MDSPI16 increased (100 ng/mL to 800 ng/mL), the hemolysis rate did not increase significantly and was less than 10% in both groups. The cytotoxic effects of the elastase inhibitor mPEG-SPA-MDSPI16 on RAW 264.7 cells, NR8383 cells, HEK293 cells, and mouse neutrophils were examined. The results are shown in Figure 2. The cytotoxicity of mPEG-SPA-MDSPI16 was consistent with its hemolytic activity. The survival rates of RAW 264.7 cells, NR8383 cells, HEK293 cells, and mouse neutrophils were all 100% at the measured concentrations (100 ng/mL to 800 ng/mL), and no cytotoxicity was observed. In addition, the results of the zebrafish embryonic toxicity test are shown in Figure S3B. After being treated with 200 ng/mL, 400 ng/mL, and 800 ng/mL of mPEG-SPA-MDSPI16, the zebrafish did not exhibit any toxic symptoms. However, in the positive control group, the zebrafish (200 μg/mL sodium dehydroacetate) exhibited cardiac bleeding, and the bodies were curved. These results show that mPEG-SPA-MDSPI16 is safe and nontoxic.

### 2.5. Immunogenic Analysis

**Figure 3:**
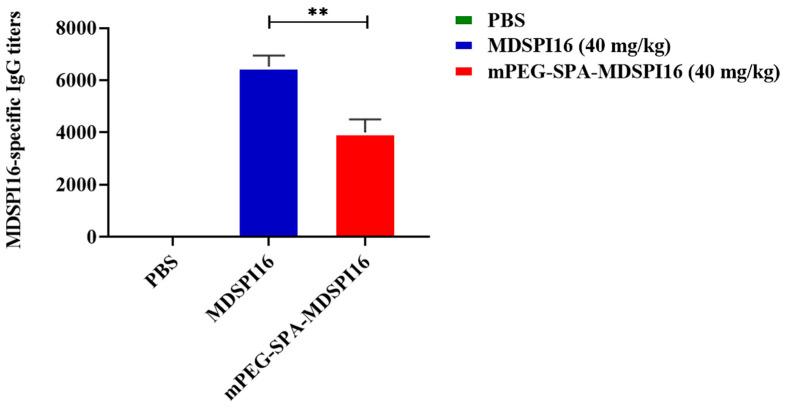
Immunogenicity evaluation of mPEG-SPA-MDSPI16. Data represented as the mean ± SEM; n = 10/group. ** *p* < 0.01 vs. the MDSPI16 (40 mg/kg) treatment group. *p*-Values were determined using an unpaired two-tailed Student’s *t*-test.

MDSPI16- and mPEG-SPA-MDSPI16-specific IgG titers were determined by ELISA. As shown in Figure 3, MDSPI16 and mPEG-SPA-MDSPI16 antibody titers were 6500 and 4000, respectively. After PEG modification, the mPEG-SPA-MDSPI16 antibody titer decreased to 62.5% of that of MDSPI16. The results show that PEG modification of MDSPI16 reduces its immunogenicity.

### 2.6. Pharmacokinetic Analysis

**Figure 4:**
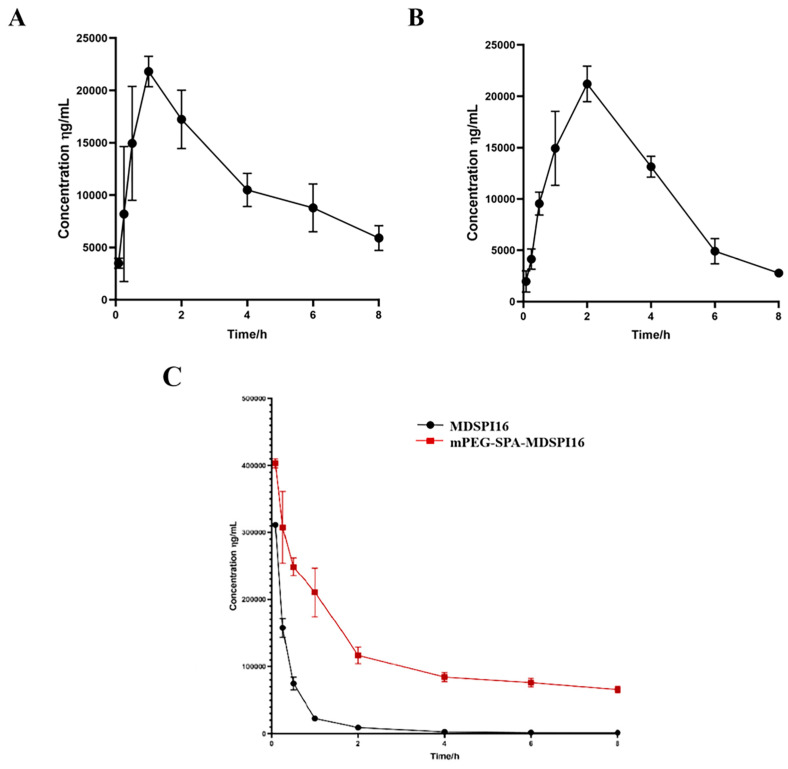
Change curve of the blood concentration of MDSPI16 with time after administration through different routes. (**A**) Changes in MDSPI16 concentration in vivo with time after subcutaneous administration. (**B**) Changes in MDSPI16 concentration in vivo with time after intramuscular administration. (**C**) Changes in MDSPI16 and mPEG-SPA-MDSPI16 concentrations in vivo with time after intravenous administration. Graphs show the mean of five biological replicates; *p*-values were determined using an unpaired two-tailed Student’s *t*-test.

The pharmacokinetic study results showed that the half-life of MDSPI16 slightly differed when it was administered through different routes (Figure 4). The plasma half-lives after subcutaneous injection, intramuscular injection, and caudal vein injection of MDSPI16 were 1.79 h, 4.08 h, and 4.33 h, respectively (Tables S1–S3). Based on this result, in this study, intravenous administration was chosen to measure the half-life of mPEG-SPA-MDSPI16 in vivo. After caudal vein injection, the plasma half-life of mPEG-SPA-MDSPI16 was extended from 4.33 h to 7.1 h compared with that of MDSPI16.

### 2.7. mPEG-SPA-MDSPI16 Ameliorated LPS-Induced Lung Inflammation and Physiological Changes in Mice

**Figure 5:**
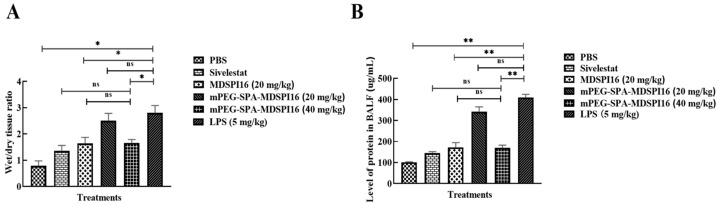
mPEG-SPA-MDSPI16 reduced the permeability of lung tissue. (**A**) W/D ratio of lung tissue. (**B**) Total protein concentration. Data represented as the mean ± SEM; n = 20/group. * *p* < 0.05, ** *p* < 0.01, ns, *p* > 0.05 vs. the LPS-treated group; ns, *p* > 0.05 vs. the mPEG-SPA-MDSPI16 (40 mg/kg) treatment group. *p*-Values were determined using an unpaired two-tailed Student’s *t*-test.

The mouse model of acute lung injury was established through a tracheal infusion of LPS, and then the therapeutic effect of mPEG-SPA-MDSPI16 was assessed by measuring body weight loss, estimating W/D ratios, and examining BALF parameters. The experimental schematic of acute lung injury in mice is shown in Figure S4. The body weight was measured after LPS stimulation. After LPS (5 mg/kg) was injected into the mice in the model group, their hair grew longer and darkened, their activity level was reduced, their response to external stimuli was weakened, their diet and water intake were reduced, and their weights were reduced. After 24 h of treatment with different doses of mPEG-SPA-MDSPI16, LPS-induced weight loss was significantly alleviated and the status of the mice was significantly improved (Figure S5). In addition, the W/D ratio of the lung tissue and the total protein concentration in BALF are shown in Figure 5. Compared with those in the phosphate-buffered saline (PBS) group, the W/D ratio and total protein concentration in the LPS group were significantly increased (*p* < 0.05 or *p* < 0.01). After treatment with mPEG-SPA-MDSPI16 (40 mg/kg), the W/D ratio and the total protein concentration in the lung tissue were significantly decreased (*p* < 0.05 or *p* < 0.01). These findings show that mPEG-SPA-MDSPI16 can effectively ameliorate pulmonary edema and reduce the permeability of lung tissue. Next, we investigated the effect of mPEG-SPA-MDSPI16 on the total number of BALF cells and neutrophils induced by LPS. The cells in BALF were stained with Wright–Giemsa, as shown in Figure S6. Compared with those in the PBS control group, the total cell numbers in BALF were significantly increased after endotracheal infusion of LPS (*p* < 0.01) and the total cell counts in the 40 mg/kg mPEG-SPA-MDSPI16 group were significantly decreased (*p* < 0.05), and there was no significant difference in the total cell counts compared with those in the MDSPI16 and Sivelestat groups (*p* > 0.05), as shown in Figure S6A. In the BALF of the PBS control group, the neutrophil count was decreased and the LPS-induced number of neutrophils increased significantly (*p* < 0.01), and after drug treatment, the neutrophil numbers in the 40 mg/kg mPEG-SPA-MDSPI16 group decreased significantly (*p* < 0.01). Compared with those in the MDSPI16 and Sivelestat groups, the neutrophil numbers in the 40 mg/kg mPEG-SPA-MDSPI16 group were not significantly different (*p* > 0.05). In conclusion, mPEG-SPA-MDSPI16 significantly reduced the total cell and neutrophil counts in the BALF of mice with ALI. Furthermore, elastase in mouse BALF was examined, and the expression of neutrophil elastase was significantly increased by LPS stimulation (*p* < 0.01). When 400 mg/kg mPEG-SPA-MDSPI16 was added, neutrophil elastase expression was significantly decreased (*p* < 0.01), but there was no significant difference compared with that in the MDSPI16 and Sivelestat groups (*p* > 0.05) (Figure S7). Taken together, these results indicate that mPEG-SPA-MDSPI16 can inhibit the inflammatory response by inhibiting neutrophil elastase activity.

### 2.8. mPEG-SPA-MDSPI16 Attenuated LPS-Induced Inflammatory Cytokine Levels in Lung Tissue and BALF Samples

**Figure 6:**
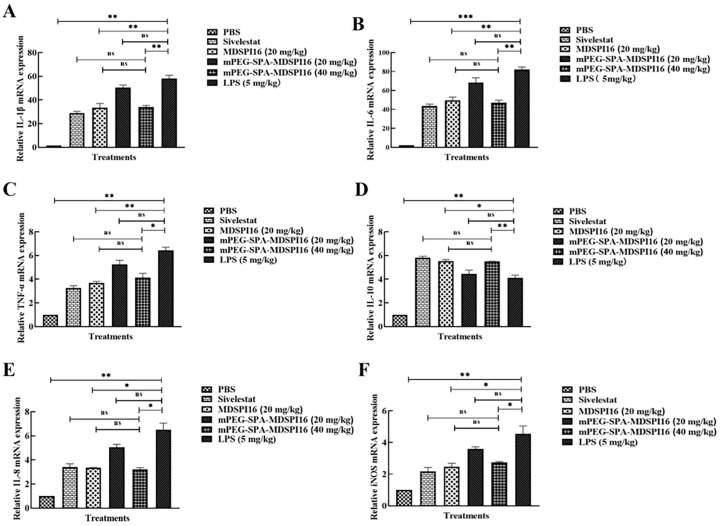
mPEG-SPA-MDSPI16 attenuated LPS-induced inflammatory cytokines in lung tissue homogenates. mPEG-SPA-MDSPI16 treatment inhibited (**A**) IL-1β, (**B**) IL-6, (**C**) TNF-α, (**E**) IL-8, and (**F**) iNOS levels in the lung tissue of mice. (**D**) mPEG-SPA-MDSPI16 treatment can increase the level of IL-10 in the lung tissue of mice. Data represented as the mean ± SEM; n = 20/group. * *p* < 0.05, ** *p* < 0.01, *** *p* < 0.001, ns, *p* > 0.05 vs. the LPS-treated group; ns, *p* > 0.05 vs. the mPEG-SPA-MDSPI16 (40 mg/kg) treatment group. *p*-Values were determined using an unpaired two-tailed Student’s *t*-test.

The mRNA levels of IL-1β, IL-6, TNF-α, IL-1β, IL-10, IL-8, and iNOS in LPS-challenged mouse lung tissue treated with a range of mPEG-SPA-MDSPI16 concentrations were analyzed by qRT–PCR. The results showed that the mRNA expression levels of IL-6, IL-1β, TNF-α, IL-8, and iNOS were significantly increased after LPS stimulation compared with those in the PBS negative control group (*p* < 0.01 or *p* < 0.001). After 40 mg/kg mPEG-SPA-MDSPI16 treatment, the mRNA expression levels of proinflammatory factors, such as IL-6, IL-1β, TNF-α, IL-8, and iNOS, were significantly decreased in a dose-dependent manner (*p* < 0.01 or *p* < 0.05), and the expression level of the anti-inflammatory factor IL-10 was significantly increased (*p* < 0.01). Moreover, there was no significant difference in the expression of inflammatory factors in the 40 mg/kg mPEG-SPA-MDSPI16 group compared with that in the MDSPI16 and Sivelestat groups (*p* > 0.05; Figure 6). Subsequently, we further examined IL-1β, IL-6, TNF-α, and IL-10 expression in mouse BALF by ELISA and observed that IL-6, IL-1β, and TNF-α expression levels were significantly increased by LPS stimulation (*p* < 0.01 or *p* < 0.001). After mPEG-SPA-MDSPI16 treatment, IL-6, IL-1β, and TNF-α expression levels were significantly decreased in a dose-dependent manner (*p* < 0.01 or *p* < 0.05). However, IL-10 expression was significantly increased (*p* < 0.01). Moreover, there was no significant difference in the expression of inflammatory factors in the 40 mg/kg mPEG-SPA-MDSPI16 group compared with that in the MDSPI16 and Sivelestat groups (*p* > 0.05; Figure S8). These results indicate that mPEG-SPA-MDSPI16 can effectively treat LPS-induced acute lung injury.

### 2.9. mPEG-SPA-MDSPI16 Ameliorated LPS-Induced Pathological Changes in Lung Tissues

**Figure 7:**
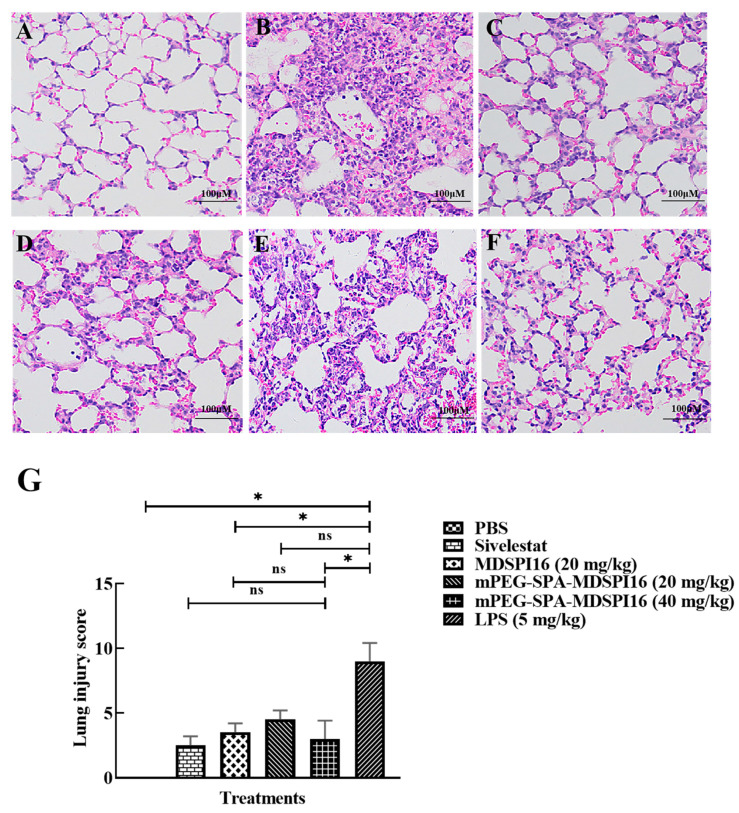
mPEG-SPA-MDSPI16 treatment ameliorates LPS-induced pathological changes in lung tissue (×200). (**A**) PBS control group. (**B**) LPS group (5 mg/kg). (**C**) Sivelestat group. (**D**) MDSPI16 group (20 mg/kg). (**E**) mPEG-SPA-MDSPI16 group (20 mg/kg). (**F**) mPEG-SPA-MDSPI16 group (40 mg/kg). (**G**) Lung injury score. Data represented as the mean ± SEM; n = 20/group. * *p* < 0.05, ns, *p* > 0.05 vs. the LPS-treated group; ns, *p* > 0.05 vs. the mPEG-SPA-MDSPI16 (40 mg/kg) treatment group. *p*-Values were determined using an unpaired two-tailed Student’s *t*-test.

We investigated the ability of mPEG-SPA-MDSPI16 to protect against LPS-induced pathological changes in mouse lung tissue by HE staining. As shown in Figure 7A–F, HE staining revealed that the alveolar wall in the PBS control group was thin and complete, the alveolar cavity was not exudated, the alveolar and pulmonary interstitial areas were not edematous, and no inflammatory cell infiltration was observed. However, in the LPS group, the pulmonary alveolar space was smaller, the alveolar space became wider, and there was substantial inflammatory cell infiltration and red blood cell exudation associated with the pathological changes in acute lung injury. After treatment with different doses of mPEG-SPA-MDSPI16, inflammatory cell infiltration in the alveolar space was significantly reduced and the alveolar structure was normalized in the LPS group compared with that in the PBS control group. Moreover, the LPS-induced increase in the lung injury score was significantly decreased by mPEG-SPA-MDSPI16 (40 mg/kg) (*p* < 0.05; Figure 7G). The MPO activity in lung tissue is shown in Figure S9. Compared with that in the PBS control group, the MPO activity in lung tissue in the LPS group was significantly increased (*p* < 0.01). After treatment with mPEG-SPA-MDSPI16, the MPO activity in lung tissue in the LPS group was significantly decreased in a dose-dependent manner (*p* < 0.05), and the MPO activity was effectively inhibited in the LPS group on treatment with 40 mg/kg of mPEG-SPA-MDSPI16 compared with that in the MDSPI16 and Sivelestat groups. Moreover, there was no significant difference in the inhibitory activity (*p* > 0.05; Figure S9).

### 2.10. mPEG-SPA-MDSPI16 Reversed the LPS-Induced Inflammatory Response in Mouse Bone Marrow Neutrophils

**Figure 8:**
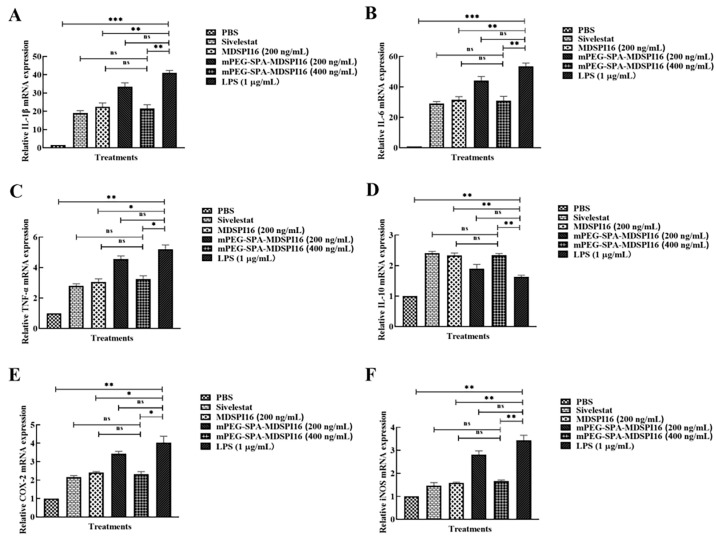
mPEG-SPA-MDSPI16 reduces the inflammatory cytokine levels in neutrophils. The cells were induced by LPS and then cultured at different concentrations of mPEG-SPA-MDSPI16 (200, 400 ng/mL). The cell pellets were collected and subjected to q-PCR for (**A**) IL-1β, (**B**) IL-6, (**C**) TNF-α, (**D**) IL-10, (**E**) COX-2, and (**F**) iNOS. Data represented as the mean ± SEM; n = 20/group. * *p* < 0.05, ** *p* < 0.01, *** *p* < 0.001, ns, *p* > 0.05 vs. the LPS-treated group; ns, *p* > 0.05 vs. the mPEG-SPA-MDSPI16 (400 ng/mL) treatment group. *p*-Values were determined using an unpaired two-tailed Student’s *t*-test.

The levels of IL-1β, IL-6, TNF-α, IL-10, COX-2, and iNOS in LPS-challenged neutrophils treated with various mPEG-SPA-MDSPI16 concentrations were analyzed by qRT–PCR. The results showed that the expression levels of IL-6, IL-1β, TNF-α, COX-2, and iNOS were significantly increased by LPS stimulation (*p* < 0.01 or *p* < 0.001). After mPEG-SPA-MDSPI16 treatment, IL-6, IL-1β, TNF-α, COX-2, and iNOS expression levels were significantly decreased in a dose-dependent manner (*p* < 0.01 or *p* < 0.05). However, IL-10 expression was significantly increased (*p* < 0.01). There was no significant difference in the expression of inflammatory factors in the 400 ng/mL mPEG-SPA-MDSPI16 group compared with that in the MDSPI16 group or the Sivelestat group (*p* > 0.05; Figure 8). IL-1β, IL-6, TNF-α, and IL-10 levels in the cell supernatant were examined by ELISA. As shown in Figure S10, the levels of IL-6, IL-1β, and TNF-α secreted by mouse neutrophils were significantly increased by LPS stimulation (*p* < 0.01). After 400 ng/mL mPEG-SPA-MDSPI16 treatment, IL-6, IL-1β, and TNF-α levels were significantly decreased (*p* < 0.01 or *p* < 0.05). However, the IL-10 level was significantly increased (*p* < 0.01). There was no significant difference in the expression of inflammatory factors between the 400 ng/mL mPEG-SPA-MDSPI16 group and the Sivelestat group (*p* > 0.05). Next, we measured the effect of mPEG-SPA-MDSPI16 on ROS production in mouse bone marrow neutrophils. The levels of ROS produced by LPS-induced neutrophils are shown in Figure S11A. Compared with neutrophils in the PBS control group, LPS-stimulated neutrophils released more ROS (*p* < 0.001). mPEG-SPA-MDSPI16 decreased the release of ROS from neutrophils in a concentration-dependent manner (*p* < 0.01). There was no significant difference in the ROS levels between the 400 ng/mL mPEG-SPA-MDSPI16, MDSPI16, and Sivelestat groups (*p* > 0.05). In addition, the neutrophil elastase levels were significantly increased after LPS stimulation compared with those in the PBS control group (*p* < 0.001). When a final concentration of 400 ng/mL mPEG-SPA-MDSPI16 was added, the expression level of neutrophil elastase was significantly decreased (*p* < 0.01) and there was no significant difference compared with the expression level of neutrophil elastase in the MDSPI16 and Sivelestat groups (*p* > 0.05; Figure S11B).

### 2.11. Effect of mPEG-SPA-MDSPI16 on the NF-κB Signaling Pathway

**Figure 9:**
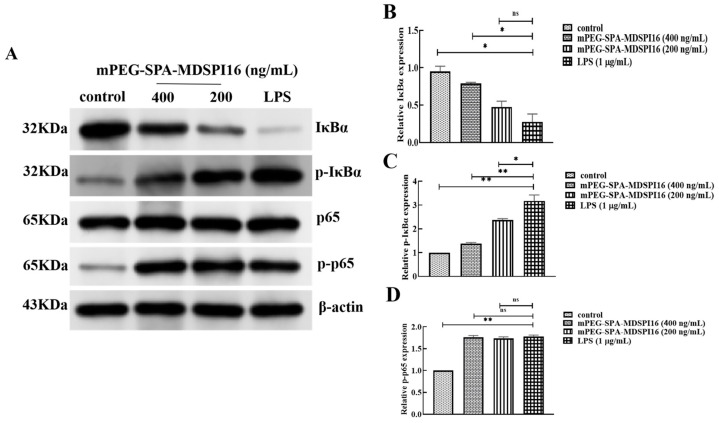
mPEG-SPA-MDSPI16 attenuated the LPS-induced inflammatory pathway through modulating the NF-κB pathways. (**A**) Representative images of Western blot. (**B**) IκBα group. (**C**) p-IκBα group. (**D**) p-p65 group; β-actin served as the internal control. Data represented as the mean ± SEM; n = 10/group. * *p* < 0.05, ** *p* < 0.01, ns, *p* > 0.05 vs. the LPS-treated group. *p*-Values were determined using an unpaired two-tailed Student’s *t*-test.

To investigate the effect of mPEG-SPA-MDSPI16 on LPS-induced activation of the NF-κB signaling pathway in neutrophils, Western blotting was used to determine the effect of LPS on IκBα protein expression and phosphorylation in neutrophils. As shown in Figure 9, the expression level of IκBα protein was significantly reduced after stimulation of cells by LPS compared with the control group (*p* < 0.05) and the expression level of IκBα protein was significantly higher in the 400 ng/mL mPEG-SPA-MDSPI16 administration group than in the LPS group (*p* < 0.05). In addition, IκBα protein phosphorylation levels increased significantly after LPS stimulation (*p* < 0.01) and decreased significantly after treatment with mPEG-SPA-MDSPI16 (200 ng/mL or 400 ng/mL) (*p* < 0.05 or *p* < 0.01). Moreover, the p65 protein expression and phosphorylation levels were not significantly different from those in the PBS control group after LPS stimulation. Compared with the control group, the expression level of p65 protein was basically unchanged after LPS stimulation (*p* > 0.05) and the phosphorylation level of p65 protein was significantly increased (*p* < 0.01). However, there was no significant difference in the p65 protein and phosphorylation levels in the mPEG-SPA-MDSPI16 group compared with those in the LPS stimulation group (*p* > 0.05). In conclusion, LPS can promote the degradation of IκBα, the phosphorylation of IκBα, and NF-κBp65 protein expression, and mPEG-SPA-MDSPI16 can inhibit the degradation of IκBα and the phosphorylation of IκBα in mouse neutrophils but has no effect on p65 protein expression or phosphorylation.

### 2.12. Effect of mPEG-SPA-MDSPI16 on the MAPK Signaling Pathway

**Figure 10:**
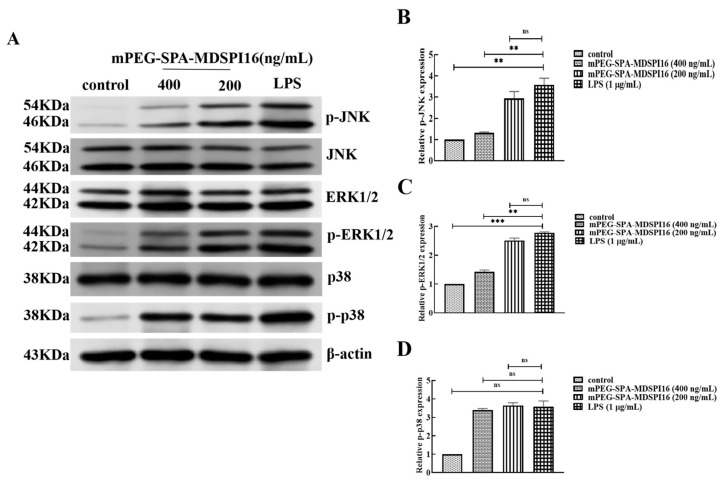
mPEG-SPA-MDSPI16 attenuated the LPS-induced inflammatory pathway through modulating the MAPK pathways. (**A**) Representative images of Western blot. (**B**) p-JNK group. (**C**) p-ERK1/2 group. (**D**) p-p38 group; β-actin served as the internal control. Data represented as the mean ± SEM; n = 10/group. ** *p* < 0.01, *** *p* < 0.001, ns, *p* > 0.05 vs. the LPS-treated group. *p*-Values were determined using an unpaired two-tailed Student’s *t*-test.

The effects of mPEG-SPA-MDSPI16 on JNK, p38, and ERK1/2 proteins and their phosphorylation levels after LPS activation of mouse neutrophils were examined by Western blotting. After LPS stimulation, the protein levels of JNK, p38, and ERK1/2 were not significantly changed. However, their phosphorylation levels were significantly increased. The phosphorylation levels of JNK and ERK1/2 in the mPEG-SPA-MDSPI16 administration group were significantly lower than those in the LPS stimulation group (*p* < 0.01), and p38 protein expression and phosphorylation levels in the mPEG-SPA-MDSPI16 administration group were not significantly different from those in the LPS stimulation group (*p* > 0.05; Figure 10). The results show that LPS can promote the phosphorylation of JNK, p38, and ERK1/2 in activated mouse neutrophils; mPEG-SPA-MDSPI16 can inhibit the phosphorylation of JNK and ERK1/2 in activated neutrophils; and mPEG-SPA-MDSPI16 has no effect on the expression or phosphorylation of p38 in activated neutrophils.

## 3. Discussion

With the progression of COVID-19 worldwide, the serine protease inhibitor Sivelestat has become an effective treatment for COVID-19-induced ALI/ARDS by improving the damage to the alveolar epithelium and vascular endothelium through inhibiting the activity of NE and reducing lung tissue damage by reversing neutrophil-mediated vascular permeability [14]. The anti-inflammatory activity of MDSPI16, which is a new serine protease inhibitor obtained from previous housefly screening, is comparable to that of Sivelestat. However, due to the large molecular weight and short half-life of this protein, it cannot be used clinically. To solve this problem, molecular modification of the serine protease inhibitor MDSPI16 was carried out using mPEG-SPA (MW = 5 kDa) modification because the structural surface of MDSPI16 contains several lysine residues. mPEG-SPA-MDSPI16, which has a long half-life and good stability, was obtained by purification with SP-Sepharose FF and RP-HPLC. The anti-inflammatory effect of mPEG-SPA-MDSPI16 was analyzed in vitro and in vivo.

The analysis of pharmaceutical properties is an important means to evaluate whether drugs can be used as clinical treatments. Therefore, we evaluated the stability and safety of mPEG-SPA-MDSPI16. The influence of temperature and pH on the inhibitory activity of mPEG-SPA-MDSPI16 was examined, and compared with MDSPI16, mPEG-SPA-MDSPI16 retained 60% inhibitory activity after incubation at 100 °C for 165 min, and mPEG-SPA-MDSPI16 still had 30% inhibitory activity after storage at 4 °C for 70 days. These findings indicate that the modified mPEG-SPA-MDSPI16 has superior temperature stability, which is conducive to the storage and transport of mPEG-SPA-MDSPI16. In addition, this study revealed that mPEG-SPA-MDSPI16 retains more than 90% of its inhibitory activity in a strongly acidic environment (pH 2.0) and that mPEG-SPA-MDSPI16 can retain 12% of its inhibitory activity in a strongly alkaline environment. These results are similar to those of PEGylated GPx1M reported by Wang et al. [15] and PEGylated recombinant phospholipase C reported by Fang et al. [16], who examined temperature and acid–base stability, respectively. Furthermore, to evaluate the in vitro and in vivo safety of mPEG-SPA-MDSPI16, this study tested the cytotoxicity, hemolytic activity, and embryotoxicity of mPEG-SPA-MDSPI16. Within the concentration range tested, neither MDSPI16 nor mPEG-SPA-MDSPI16 exhibited hemolytic activity, cytotoxicity, or embryotoxicity. This finding indicates that mPEG-SPA-MDSPI16 retains the safety of the unmodified protein MDSPI16 after PEG modification. In conclusion, mPEG-SPA-MDSPI16 has good stability and safety and the potential to be used in clinical practice.

Immunogenicity is an important index of macromolecular protein drugs to evaluate. Foreign proteins can easily trigger an immune response, and the higher the molecular weight, the stronger the immunogenicity. Previous studies have shown that PEG can mask the antigenic determinants on the protein surface and reduce the immunogenicity of a protein through covalent binding [17]. In this study, the immunogenicities of MDSPI16 and mPEG-SPA-MDSPI16 were measured, and the antibody titer of MDSPI16 was decreased by 62.5% compared with the antibody titer in the other group. In addition to immunogenicity, another major factor limiting the clinical use of protein peptides is their short half-lives, which results in greatly reduced drug bioavailability. The half-lives of MDSPI16 and mPEG-SPA-MDSPI16 in rat plasma showed that the plasma half-life of MDSPI16 administered by intravenous injection was the longest and the half-life of mPEG-SPA-MDSPI16 administered by intravenous injection was extended from 4.33 h to 7.1 h. These results are consistent with those of Hyeran et al. [18], who used dTCTP-binding peptide 2 (dTBP2) PEGylation to prolong the plasma half-life of PEG-dTBP2 by approximately 2.5-fold in mice and significantly reduced the migration of inflammatory cells and the levels of cytokines in bronchoalveolar lavage fluid. Compared with that of MDSPI16, the immunogenicity of mPEG-SPA-MDSPI16 was significantly reduced and the in vivo half-life was prolonged. These results show that mPEG-SPA-MDSPI16 can become a drug for use in vivo.

To evaluate the effect of mPEG-SPA-MDSPI16 on LPS-induced ALI, we established a mouse ALI model, and after treatment with mPEG-SPA-MDSPI16, lung tissue edema was significantly reduced, the mental state of the mice was improved, and the survival rate was significantly increased. In addition, pathological observations revealed that a large number of neutrophils migrated to the lung tissue and participated in inflammation. By examining total cells, neutrophils, and neutrophil elastase activity in BALF, it was initially confirmed that mPEG-SPA-MDSPI16 could reduce the number of neutrophils at the site of inflammation by inhibiting the release of elastase, which indicates that mPEG-SPA-MDSPI16 can inhibit the migration of neutrophils to lung tissue and reduce inflammatory damage in lung tissue. Furthermore, studies have confirmed that excessive release of cytokines by activated neutrophils can cause tissue damage and that protease inhibitors can reduce the inflammatory response of cells by inhibiting the release of cytokines [19]. For example, urinary trypsin inhibitors exert clinical effects on ARDS patients by inhibiting the release of cytokines, such as IL-8 and TNF-α [20]. The protease inhibitor ulinastatin can reduce the expression of the proinflammatory factors IL-6, IL-8, and TNF-α, thereby improving the severity of sepsis in the urinary system in elderly individuals [21]. In a cellular inflammatory model of LPS-induced neutrophil activation, the elastase inhibitor Civilax sodium effectively inhibited inflammation caused by neutrophil activation by inhibiting the expression of the cytokines IL-6, IL-1β, and TNF-α [22]. The serine protease inhibitor mPEG-SPA-MDSPI16, which is a highly active elastase inhibitor, can significantly promote the expression and release of the anti-inflammatory factor IL-10 in LPS-induced mouse lung tissue and BALF and inhibit the expression and release of the proinflammatory factors IL-6, IL-1β, TNF-α, and IL-8. This study examined the expression levels of iNOS and COX-2 to determine the role of mPEG-SPA-MDSPI16 in the inflammatory response after neutrophil activation. The results showed that the expression levels of iNOS and COX-2 were significantly increased after neutrophil overactivation, and large amounts of NO and PGS were released, causing tissue damage. mPEG-SPA-MDSPI16 could significantly inhibit the expression of iNOS and COX-2, thereby reducing the degree of inflammation. In conclusion, mPEG-SPA-MDSPI16 has a good therapeutic effect against LPS-induced ALI and has the potential to be a new anti-inflammatory drug.

To assess the mechanism by which mPEG-SPA-MDSPI16 can treat ALI, we established an LPS-induced neutrophil inflammation model and found that mPEG-SPA-MDSPI16 can significantly reduce the production of neutrophil ROS in mice and significantly reduce the expression of IL-6, TNF-α, IL-1β, and other proinflammatory factors. These results further prove that mPEG-SPA-MDSPI16 can treat ALI in mice by inhibiting neutrophil elastase activity, preventing neutrophil overactivation, and reducing the expression and release of inflammatory mediators. The elastase inhibitor Sivelestat can effectively inhibit the accumulation of neutrophils and the release of TNF-α, IL-6, and MPO in the bronchoalveolar lavage fluid of rats and can alleviate LPS-induced ALI by decreasing the expression of NF-κB p65 [23]. In this study, the effect of the elastase inhibitor mPEG-SPA-MDSPI16 on the NF-κB signal transduction pathway was investigated. The results showed that mPEG-SPA-MDSPI16 can significantly inhibit the phosphorylation and degradation of IκBα. However, mPEG-SPA-MDSPI16 had no significant effects on the expression of p65. We hypothesized that mPEG-SPA-MDSPI16 plays an inhibitory role in the preactivation of the NF-κB signaling pathway. The MAPK pathway consists of three classical signal transduction pathways: the c-Jun N-terminal kinase (JNK) signaling pathway, the p38 MAPK signaling pathway, and the extracellular signal-regulated kinase (ERK) signaling pathway [24,25,26]. The activated MAPK pathway is involved in various activities, such as the stress response, signal transduction, apoptosis, and the development of inflammatory disease, by regulating the activity of various transcription factors [27]. Therefore, the effect of the elastase inhibitor mPEG-SPA-MDSPI16 on the MAPK signal transduction pathway was also investigated in this study. The results showed that the elastase inhibitor mPEG-SPA-MDSPI16 can inhibit the activation of the JNK and ERK1/2 signaling pathways by inhibiting the phosphorylation of JNK and ERK1/2. These findings show that mPEG-SPA-MDSPI16 may be a novel JNK signaling pathway blocker and play an important anti-inflammatory role in the neutrophil inflammatory response.

## 4. Materials and Methods

### 4.1. Chemicals and Reagents

The chemicals/reagents used in this study and the company they were purchased from are listed next. Mouse bone marrow neutrophils (C57BL/6 cells) were purchased from Charles River Labs (L’Arbresle, France). mPEG-succinimidyl propionate with a molecular weight of 5 kDa (mPEG-SPA) was obtained from Jenkem Biotech (Beijing, China). LPS (isolated from *Escherichia coli* strain 055: B5), CCK-8 assay reagent, acetonitrile (ACN), and Dulbecco’s modified Eagle medium (DMEM) were obtained from Sigma (St. Louis, MO, USA). Luria–Bertani (LB) broth was purchased from Sangon Biotech (Shanghai, China).

### 4.2. Animals

BABL/c mice (weighing 20 ± 2 g) and Wistar rats (weighing 300–320 g) were purchased from the Center of Experimental Animals of the Changchun Institute of Biological Products (license key: SCXK J 2016-0008), housed with commercial food and water, and exposed to a 12 h/12 h light/dark cycle. The laboratory animal usage license (SYXK-2018-0023) was issued by the Laboratory Animal Center of Jilin Agricultural University Changchun, China. All animal studies (including the mice euthanasia procedure) were performed in compliance with the regulations and guidelines of the Institutional Animal Care of Jilin Agricultural University Changchun, China.

### 4.3. Modification and Purification of mPEG-SPA-MDSPI16

MDSPI16 was obtained from our previous study [28]. mPEG-SPA and MDSPI16 were mixed with 1 mL of 50 mM phosphate buffer (pH 6.4) in a tube, the final concentration of MDSPI16 was 1.9 mg/mL, and the molar mass ratio of mPEG-SPA to MDSPI16 was 42.8. mPEG-SPA and MDSPI16 were stirred at 100 rpm/min for 1 h at 25 °C, after which the reaction mixture was placed into a dialysis bag with a molecular weight cut-off of 10 kDa to remove the unreacted mPEG-SPA at 4 °C. The modified products were loaded onto an SP-Sepharose FF column (strong cation exchange column; GE Healthcare, Chicago, IL, USA) connected to an AKTA purification system (Boston, MA, USA). The products were eluted with different concentrations of NaCl (50 mM, 100 mM, 200 mM, 300 mM, and 400 mM) in 20 mM sodium acetate buffer solution (pH 5.0) at a 1 mL/min flow rate. The proteins were classified according to the different polarities, and the separated peaks were collected at 280 nm and analyzed by SDS-PAGE. Next, for further purification of mPEG-SPA-MDSPI16, the modified products were applied to a reverse-phase high-performance liquid chromatography system (RP-HPLC, Agilent, Santa Clara, CA, USA) equipped with a volume exclusion column (BioCore SEC-300, 7.8 mm × 300 mm, 5 μm particle size) (Nano Chrom Technologies Co., Ltd., Suzhou, China). Iso-elution was performed with 20 mM phosphate buffer (pH 7.0) containing 300 mM NaCl and 10% acetonitrile as the mobile phase at a flow rate of 0.6 mL/min. Separate peaks of the UV detector were recorded at 280 nm. For this test, after each purification step, elastase was used as the hydrolysis target, and the in vitro biological activity of the modified product was determined by the casein plate method.

### 4.4. Temperature and pH Stability

The temperature and pH stability of mPEG-SPA-MDSPI16 were tested, as described by Tang et al. [28]. Equivalent amounts of mPEG-SPA-MDSPI16 were placed in test tubes. The test tubes were sealed and then placed at different temperatures (37 °C, 40 °C, 50 °C, 60 °C, 70 °C, 80 °C, 90 °C, 100 °C) for 15 min, at 100 °C for different durations (15, 30, 45, 60, 75, 90, 105, 120, 135, 150, 165, and 180 min), and at 4 °C for different durations (0, 10, 20, 30, 40, 50, 60, and 70 days). To determine the effect of acids and bases on the inhibitory activity of mPEG-SPA-MDSPI16, mPEG-SPA-MDSPI16 was treated with Britton–Bertani buffer at different pH values (2, 3, 4, 5, 6, 7, 8, 9, 10, 11, 12, and 13) for 24 h at room temperature. Subsequently, the temperature and pH stabilities of mPEG-SPA-MDSPI16 were analyzed by examining the inhibitory effects of different mPEG-SPA-MDSPI16 samples on elastase. For the experiment outlined before, the unmodified serine protease inhibitor MDSPI16 was used as a positive control, and the effects of mPEG-SPA-MDSPI16 were estimated by measuring the residual enzyme activity. Each experiment was repeated three times.

### 4.5. Determination of the IC_50_ of mPEG-SPA-MDSPI16

As described by Yang et al. [29], N-succinyl-Ala-ala-p-nitroanilide was used as the elastase substrate to further determine the half-maximal inhibitory concentration (IC_50_) of MDSPI16 and mPEG-SPA-MDSPI16 at the unit point. Specifically, 500 ng of the unit point-modified product was added to 100 ng of protease solution, after which 100 mM Tris-HCl (pH 7.4) was added to 100 μL of the protease solution. The resultant solution was mixed evenly and incubated at 37 °C for 30 min. Next, 100 μL of elastase substrate was added to the reaction mixture, which was incubated at 37 °C for 1 h. Absorbance was measured at 405 nm. The remaining enzyme activity was calculated according to the absorbance, and the remaining enzyme activity without the addition of the unit point-modified product was the initial reaction rate (100%). The inhibitory activity was expressed as the remaining enzyme activity relative to the initial reaction rate, and the IC_50_ required to inhibit 50% elastase hydrolysis was calculated.

### 4.6. Safety Assay

#### 4.6.1. In Vitro Safety Assay

The hemolytic activity of mPEG-SPA-MDSPI16 was evaluated, as described previously [30]. Briefly, sheep blood cells were washed five times with PBS (pH 7.4) and diluted to 2% in PBS. Next, equal volumes of sheep blood cells were mixed with different concentrations of mPEG-SPA-MDSPI16 (100 ng/mL to 800 ng/mL) in tubes and incubated at 37 °C for 1 h. Subsequently, the mixtures were centrifuged at 1000× *g* for 10 min, and the supernatants were transferred to 96-well plates. Triton X-100 (0.2%) and PBS were added as positive and negative controls, respectively. The absorbance of the mixtures was measured as the OD at 570 nm. Hemolysis was calculated using the following formula: hemolysis (%) = [(A_420, sample_ − A_420, PBS_)/(A_420, 0.2% Triton X-100_ − A_420, PBS_)] × 100. To evaluate the safety of mPEG-SPA-MDSPI16 for cells from different sources, rat alveolar macrophages (NR8383 cells), human embryonic kidney cells (HEK293 cells), mouse mononuclear macrophages (RAW264.7 cells), and mouse neutrophils were used to study the cytotoxicity of mPEG-SPA-MDSPI16 [18]. Briefly, 10^5^ cells/well (mouse neutrophils, NR8383 cells, HEK293 cells, and RAW264.7 cells) were seeded in a 96-well plate, and after 12 h, the cells were treated with various concentrations of mPEG-SPA-MDSPI16 (100 ng/mL to 800 ng/mL) or with 0.2% Triton X-100 (positive control) and PBS (negative control) in the presence of LPS (1 µg/mL) for 24 h. Meantime, a zero regulation group containing only the culture medium (blank group) and a control group containing only cells (control group) were established. Next, CCK-8 (10%, *v*/*v*) was added to each well, and the cells were cultured at 37 °C for 4 h. Subsequently, absorbance (optical density (OD)) was quantified by a plate reader (Bio-Rad) at 450 nm. The resultant viability was calculated as follows: cell viability = (OD_Treated_ − OD_Blank_)/(OD_Control_ − OD_Blank_). The experiment was performed three times.

#### 4.6.2. In Vivo Safety Assay

The developmental toxicology of zebrafish embryos was determined, as described by Song et al. [31]. Zebrafish embryos were exposed to different concentrations of MDSPI16 (200–800 ng/mL) or mPEG-SPA-MDSPI16 (200–800 ng/mL) 48 h after fertilization; 200 μg/mL of sodium dehydroacetate served as a positive control, and the embryo medium served as a negative control. After 72 h at 28 °C, phenotypic changes in the zebrafish were observed under an inverted microscope.

### 4.7. Immunogenicity Assay

The immunogenicities of MDSPI16 (40 mg/kg) and mPEG-SPA-MDSPI16 (40 mg/kg) were analyzed as previously described [32]. In brief, 40 mice were divided into 4 groups, with 10 mice in each group. MDSPI16 (40 mg/kg), mPEG-SPA-MDSPI16 (40 mg/kg), and PBS were injected intramuscularly on day 0, day 7, and day 14. On the 21st day, anesthetized (ketamine (60 mg/kg) and xylazine (8 mg/kg) (Sigma-Aldrich, Shanghai, China)) mice were orbitally bled, and blood samples were collected and centrifuged at 2000 rpm for 20 min. MDSPI16-specific IgG titers in the serum were determined by indirect enzyme-linked immunosorbent assay (ELISA).

The concentration of MDSPI16 was adjusted to 8.0 µg/mL with a coating solution (0.05 M carbonate/bicarbonate buffer, pH 9.6), 100 µL was added to each well of a 96-well plate for coating, and the plate was incubated at 4 °C overnight. Next, 200 μL of 2% powdered milk diluted in PBS (MPBS) was added to each well, and the reaction mixture was incubated at 37 °C for 2 h. The plate was washed five times with PBS and then washed five times with 0.05% PBS-Tween (PBS-T). Subsequently, the sample was diluted 2-fold with PBSM (from 1:100 to 1:12,800), the diluted serum was added to each well (100 μL/well), and the reaction mixture was incubated at 37 °C for 1 h. The plate was washed five more times, as previously described. Next, the plate was incubated with 100 μL of 5000-fold diluted IgG-HRP (anti-mouse, IgG-HRP, A9044; Sigma-Aldrich, Shanghai, China) at 37 °C for 1 h. After 1 h of incubation at 37 °C, the plate was washed three times with PBS-T and five times with PBS. One hundred microliters of OPD-H_2_O_2_ substrate (SIGMAFAST OPD tablets; Sigma-Aldrich, Shanghai, China) was added to each plate well, and the plate was incubated at 37 °C for 15 min. After the reaction was stopped by adding 50 μL of 1 M H_2_SO_4_ (Merck^®^, Shanghai, China), absorbance was measured at 490 nm with a microplate reader (Sunrise Basic Tecan, Männedorf, Switzerland). Each experiment was repeated three times.

### 4.8. A Pharmacokinetic Study in Wistar Rats

To investigate the half-lives of MDSPI16 and mPEG-SPA-MDSPI16, pharmacokinetic studies of MDSPI16 and mPEG-SPA-MDSPI16 were performed on Wistar rats (300–310 g) [33]. Wistar rats were immunized with MDSPI16 or mPEG-SPA-MDSPI16 via tail vein injection, subcutaneous injection, or intramuscular injection. Blood samples were taken at 0, 2, 4, 6, and 8 h to examine the metabolism of MDSPI16 and mPEG-SPA-MDSPI16 in vivo. With time as the horizontal coordinate and the blood drug concentration as the vertical coordinate, GraphPad Prism software (version 8.0.2.263) was used to fit the drug–time curve.

### 4.9. Induction of ALI in Mice by Intratracheal Instillation of LPS

#### 4.9.1. Mouse Model of ALI and Treatment

To study the influence of mPEG-SPA-MDSPI16 on acute lung injury (ALI), intratracheal instillation of LPS (5 mg/kg) was performed, as described by Satya et al. [34], to establish a standard model of ALI in mice. In our previous experiment, we found that male mice have better physical health indicators than female mice. Therefore, we chose male mice to conduct the experiment. Male mice were randomly assigned to six groups (n = 20/group). (1) In the PBS control (blank control) group, PBS was injected into the mice. (2) In the negative control group, LPS (5 mg/kg) was injected into the mice. (3) In the positive control group, the mice were injected with LPS (5 mg/kg) or Sivelestat (20 mg/kg). (4–6) In the drug treatment groups, the mice were injected with LPS plus mPEG-SPA-MDSPI16 (5 mg/kg LPS + 20 mg/kg MPEG-SPA-MDSPI16; 5 mg/kg LPS + 40 mg/kg mPEG-SPA-MDSPI16) or LPS plus MDSPI16 (5 mg/kg LPS + 20 mg/kg MDSPI16). LPS was dissolved in PBS to a concentration of 5 mg/mL. After the mice were anesthetized, the mice in all groups except for those in the blank control group were injected with LPS (5 mg/kg), and the mice in the positive control group were intraperitoneally injected with Sivelestat (20 mg/kg) 2 h later. In the drug treatment groups, 20 mg/kg of mPEG-SPA-MDSPI16, 40 mg/kg of mPEG-SPA-MDSPI16, or 20 mg/kg of MDSPI16 was injected. The mice in each group were weighed before and after drug administration. Myeloperoxidase (MPO) concentrations in the left lung were measured according to the instructions of the MPO test kit (Nanjing Jiancheng Bioengineering Institute, Nanjing, China).

#### 4.9.2. Lung Wet–Dry Weight (W/D) Ratio

The severity of pulmonary edema was assessed by calculating the wet-to-dry weight ratio of lung tissues [35]. Mice without BALF were sacrificed under anesthesia. Left lung tissues were excised and weighed (wet weight) after surface water and blood were blotted with absorbent paper. The samples were subsequently placed in an oven at 60 °C for 72 h, after which the dry weight was determined. The W/D ratio was calculated to assess the water content in mouse lung tissue.

#### 4.9.3. RNA Isolation and Inflammatory Factor Analysis in Lung Tissue

The expression levels of cytokines (IL-10, IL-6, IL-1β, TNF-α, IL-8, and iNOS) in mouse lung tissue were analyzed by RT–qPCR. In this study, β-actin was used as the endogenous control, PBS was added to the blank control group, LPS was added to the negative control group, and Sivelestat was used in the positive control group; each group contained three replicates. The qRT–PCR primers used are listed in Table S4. Total RNA was extracted from mouse lung tissue using RNAiso Plus, as previously described [7]. After the concentration and purity of the RNA were determined by spectrophotometry, 1 μg of RNA was reverse-transcribed to synthesize first-strand complementary DNA. A cDNA synthesis kit (TransGen, Beijing, China) was used according to the manufacturer’s instructions. Gene expression was examined using 2× PerfectStart^®^ Green qPCR SuperMix and Passive Reference Dye (50×) (TransGen, Beijing, China), and the relative levels of gene expression were analyzed by the 2^−ΔΔCT^ method.

#### 4.9.4. Histopathology

Hematoxylin–eosin (H&E) staining was used to observe the severity of pulmonary edema and lung injury in mice with alveolitis, as previously described [36]. In brief, the middle lobes of the right lung were collected, rinsed with 0.9% NaCl, fixed with 4% paraformaldehyde, and subjected to HE staining. The scoring of lung injury was performed, as previously described [37].

#### 4.9.5. Inflammatory Factor Analysis and Neutrophil Elastase Activity Analysis in Mouse BALF

After the mice were anesthetized, as previously described, a vascular catheter was inserted into the trachea, and the lung tissue was flushed 5 times with saline and then aspirated. The obtained BALF was centrifuged at 3000 rpm for 5 min, and a portion of the supernatant was used to determine the total protein concentration [38]. Cytokine expression levels (IL-10, IL-6, IL-1β, and TNF-α) in BALF supernatants were measured using ELISA kits (Nanjing Jiancheng Bioengineering Institute, Nanjing, China) according to the manufacturer’s instructions. The total numbers of cells and neutrophils in the lavage solution were measured by Reichsmard–Giemsa staining (Nanjing Jiancheng Bioengineering Institute, Nanjing, China). The experimental procedure was performed according to the instructions of the cell staining kit. The activity of neutrophil elastase in mouse BALF was measured according to the instructions of the neutrophil elastase detection kit.

### 4.10. LPS-Induced Cell Injury

#### 4.10.1. Cell Culture and Treatments

Mouse bone marrow neutrophils were cultured in DMEM containing 10% FBS at 37 °C and 5% CO_2_ until the cell density reached 85–90%. The cells were harvested by centrifugation at 1000 rpm for 5 min and subcultured in fresh medium. Next, adherent cells in the logarithmic growth phase were selected, LPS at a final concentration of 1 µg/mL was added, and the reaction mixture was incubated for 5 h. mPEG-SPA-MDSPI16 (200 ng/mL and 400 ng/mL), MDSPI16 (200 ng/mL), or Sivelestat was added, and the resulting mixture was incubated for 12 h. Subsequently, cell precipitates were collected, and cytokine (IL-10, IL-6, IL-1β, TNF-α, COX-2, and iNOS) expression levels in mouse bone marrow neutrophils were assessed by qRT–PCR. Reactive oxygen species (ROS) levels in neutrophils treated with mPEG-SPA-MDSPI16 were determined by an ROS kit (Nanjing Jiancheng Bioengineering Institute, Nanjing, China). Neutrophil supernatants were collected, and cytokine expression levels (IL-10, IL-6, IL-1β, and TNF-α) in neutrophil supernatants were measured using ELISA kits (Nanjing Jiancheng Bioengineering Institute, Nanjing, China) according to the manufacturer’s instructions. Neutrophil elastase activity in neutrophil supernatants was determined with a mouse neutrophil elastase detection kit (Nanjing Jiancheng Bioengineering Institute, Nanjing, China).

#### 4.10.2. Effect on the NF-κB and MAPK Signaling Pathways in Neutrophils

A BCA protein detection kit was used to determine the concentration of the precipitated proteins, and rabbit anti-mouse IκBα, p-IκBα, p65, p-p65, JNK, p-JNK, p38, and p-p38 primary antibodies were used. A rabbit anti-mouse β-actin antibody (Beverly, MA, USA) was used as the internal reference, a sheep anti-rabbit IgG-HRP antibody was used as the secondary antibody, and protein expression was determined by Western blotting.

### 4.11. Statistical Analysis

All the experimental data were processed by SPSS (version 22.0) using an unpaired two-tailed Student’s *t*-test. The data were expressed as the mean ± standard error of the mean (SEM). A *** *p*-value of < 0.001 was considered to indicate an extremely significant difference; ** *p*-values < 0.01 and * *p*-values < 0.05 were considered to indicate significant differences. The mean values were considered not significantly different (ns) when *p* > 0.05. The statistical results are clearly displayed in the form of graphs generated using GraphPad Prism software (version 8.0.2.263).

## 5. Conclusions

In summary, MDSPI16 was modified with mPEG-SPA (Mw = 5 kDa), the modified product mPEG-SPA-MDSPI16 was purified, and its IC_50_ for elastase was 86 nM. Studies of its pharmaceutical properties showed that mPEG-SPA-MDSPI16 has good stability and safety, low immunogenicity, and a long half-life. Studies on the underlying anti-inflammatory mechanism showed that mPEG-SPA-MDSPI16 exerts anti-inflammatory effects by inhibiting the activation of the NF-κB signaling pathway and the MAPK signaling pathway in mouse neutrophils and inhibiting the synthesis and release of the inflammatory mediators IL-6, IL-1β, TNF-α, and neutrophil elastase. In addition, mPEG-SPA-MDSPI16 has good anti-inflammatory effects on LPS-induced acute lung injury-related inflammation in mice. These results suggest that mPEG-SPA-MDSPI16 has the potential to be an anti-inflammatory drug.

## Data Availability

The datasets supporting the conclusions of this article will be made available by the authors without undue reservation.

## Supplementary Materials

The supporting information can be downloaded at: https://www.mdpi.com/article/10.3390/ijms25084567/s1.
