# Supplementary material for: The Long-Acting Serine Protease Inhibitor mPEG-SPA-MDSPI16 Alleviates LPS-Induced Acute Lung Injury
Source: Int J Mol Sci. 2024 Apr 22;25(8):4567. doi: 10.3390/ijms25084567 (PMC11049807; doi:10.3390/ijms25084567)
Supplement: Supplementary file 1 [file ijms-25-04567-s001.zip › ijms-2965002-supplementary.pdf]

International Journal of
Molecular Sciences

MDPI

Article

The long-acting serine protease inhibitor mPEG-SPA-MDSPI16 alleviates LPS-induced acute lung injury

Supplementary Figure

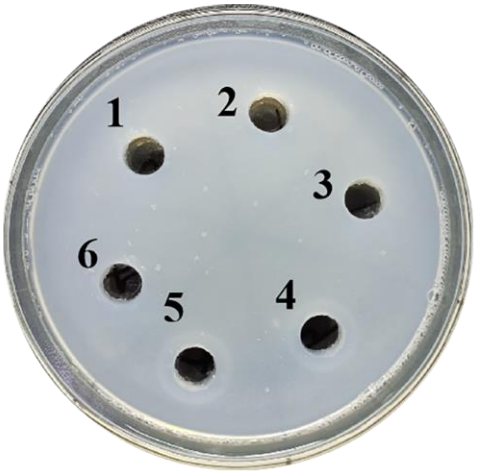

Figure S1. mPEG-SPA-MDSPI16 inhibited elastase activity. 1 MDSPI16+Elastase. 2 mPEG-SPA-MDSPI16+Elastase. 3 Sivelestat+Elastase. 4 Elastase. 5 mPEG-SPA+Elastase. 6 mPEG-SPA.

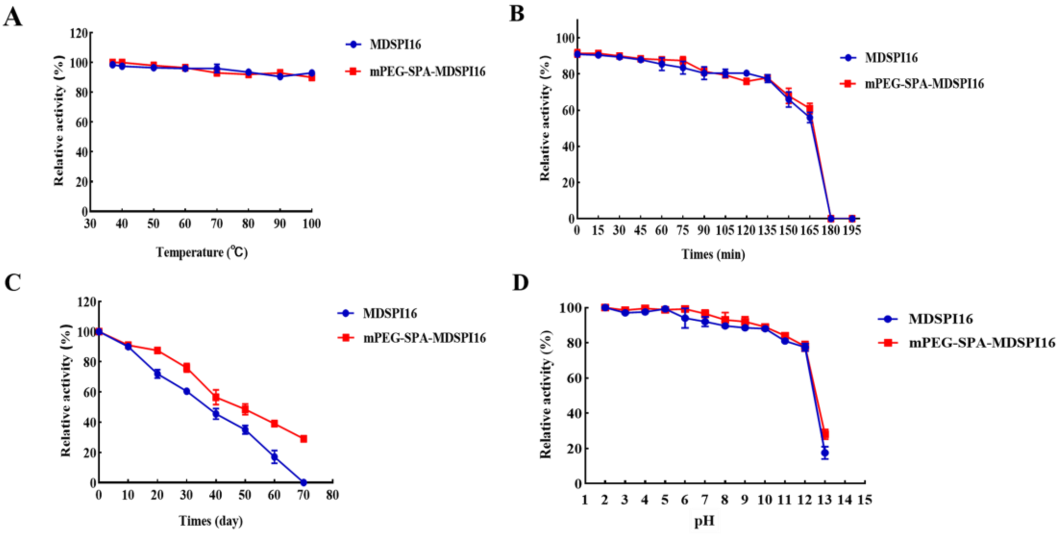

Figure S2. Stability analysis of mPEG-SPA-MDSPI16. (A, B and C) Temperature stability. (D) pH stability. Graphs show mean of three biological replicates, p-values were determined using an unpaired two-tailed Student' s t-test.

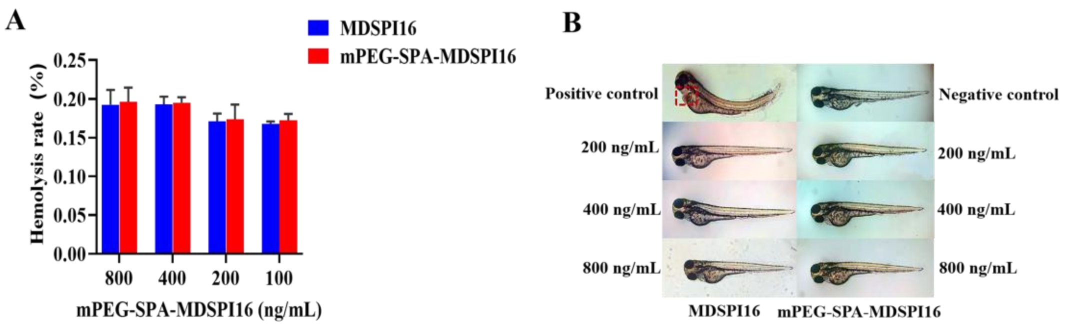

Figure S3. Determination of hemolytic activity and embryotoxicity. (A) Determination of hemolytic activity of mPEG-SPA-MDSPI16. (B) Embryotoxicity test results of mPEG-SPA-MDSPI16. Graphs show mean of three biological replicates, p-values were determined using an unpaired two-tailed Student' s t-test.

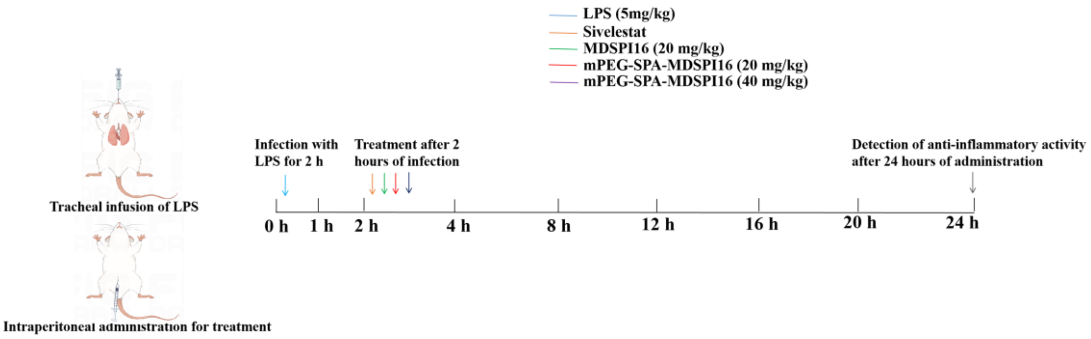

Figure S4. The experimental schematic of acute lung injury in mice. n = 20/group.

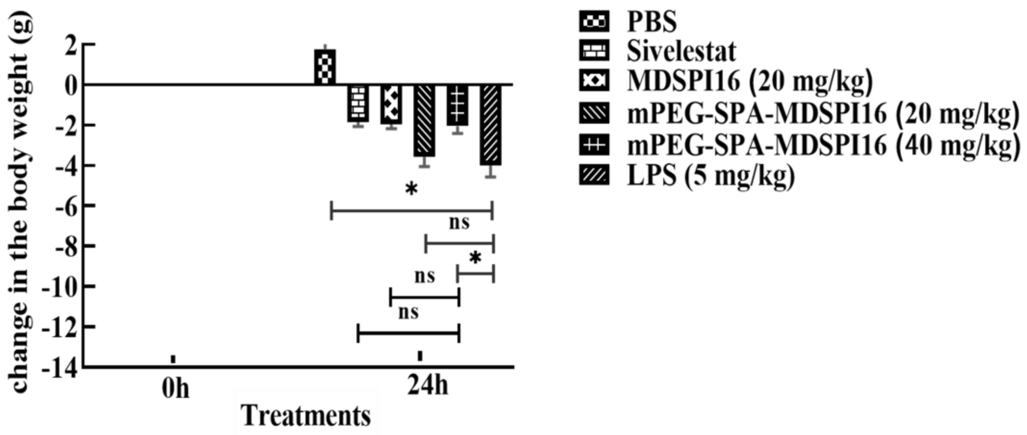

Figure S5. mPEG-SPA-MDSPI16 improved LPS-induced weight loss in Mice. Data represented as Mean ± SEM, n = 20/group. * $p < 0.05$, ns, $p > 0.05$ vs. the LPS-treated group; ns, $p > 0.05$ vs. the mPEG-SPA-MDSPI16(40 mg/kg) treatment group. P-values were determined using an unpaired two-tailed Student' s t-test.

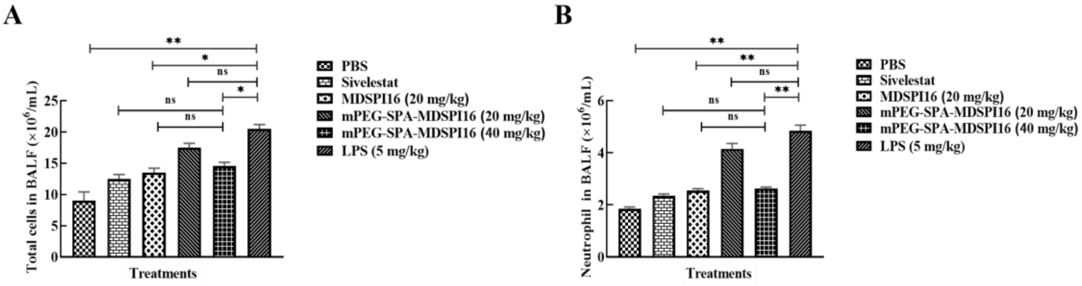

Figure S6. Cell count in mouse BALF. (A) Total cells in BALF. (B) Neutrophils in BALF. Data represented as Mean ± SEM, n = 20/group. * $p < 0.05$, ** $p < 0.01$, ns, $p > 0.05$ vs. the LPS-treated group; ns, $p > 0.05$ vs. the mPEG-SPA-MDSPI16(40 mg/kg) treatment group. P-values were determined using an unpaired two-tailed Student' s t-test.

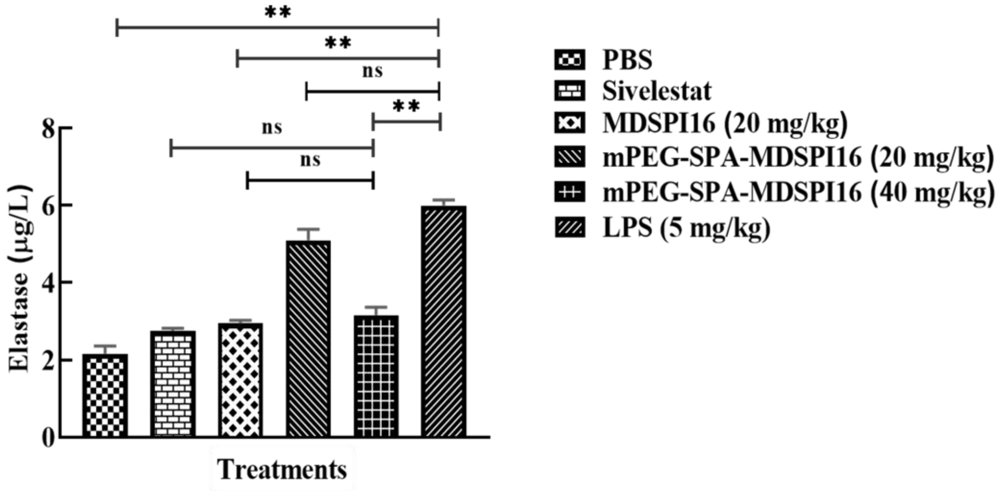

Figure S7. mPEG-SPA-MDSPI16 inhibited the expression of neutrophil elastase in mouse BALF. Data represented as Mean ± SEM, n = 20/group. ** $p < 0.01$, ns, $p > 0.05$ vs. the LPS-treated group; ns, $p > 0.05$ vs. the mPEG-SPA-MDSPI16(40 mg/kg) treatment group. P-values were determined using an unpaired two-tailed Student' s t-test.

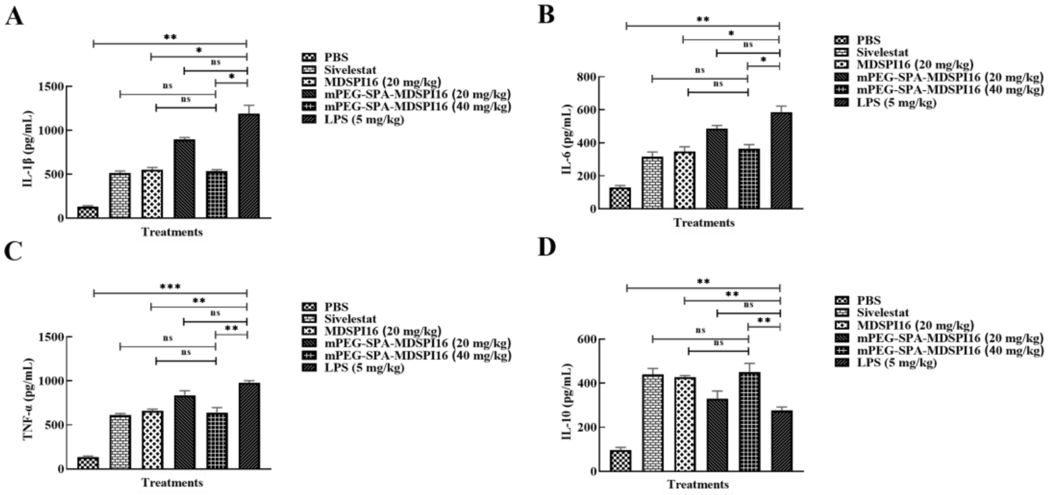

Figure S8. mPEG-SPA-MDSPI16 attenuated the LPS induced inflammatory cytokines in BALF samples. mPEG-SPA-MDSPI16 treatment inhibited the(A)IL-1β, (B) IL-6 and (C)TNF-*α* level in mice BALF. (D) mPEG-SPA-MDSPI16 treatment can increase the level of IL-10 in mice BALF. Data represented as Mean ± SEM, n = 20/group. * $p < 0.05$, ** $p < 0.01$, *** $p < 0.001$, ns, $p > 0.05$ vs. the LPS-treated group; ns, $p > 0.05$ vs. the mPEG-SPA-MDSPI16(40 mg/kg) treatment group. P-values were determined using an unpaired two-tailed Student' s t-test.

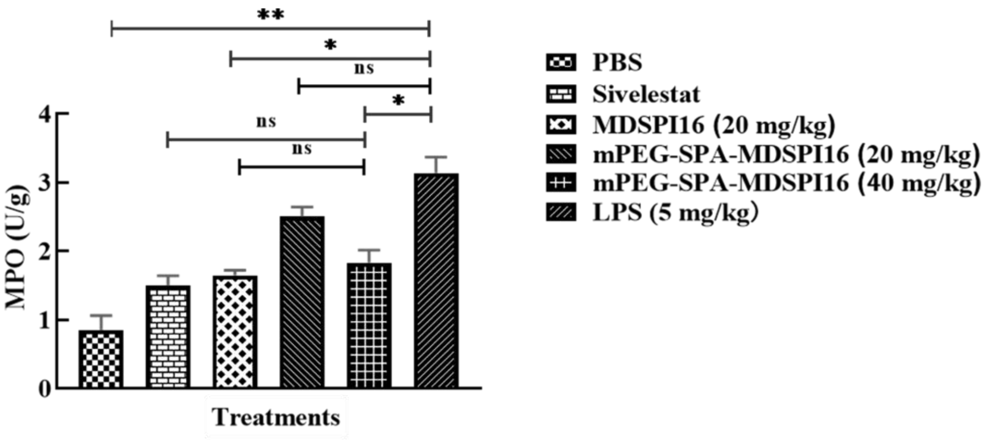

Figure S9. Quantification of MPO activity in lung tissue of ALI mice. Data represented as Mean ± SEM, n = 20/group. * $p < 0.05$, ** $p < 0.01$, ns, $p > 0.05$ vs. the LPS-treated group; ns, $p > 0.05$ vs. the mPEG-SPA-MDSPI16(40 mg/kg) treatment group. P-values were determined using an unpaired two-tailed Student' s t-test.

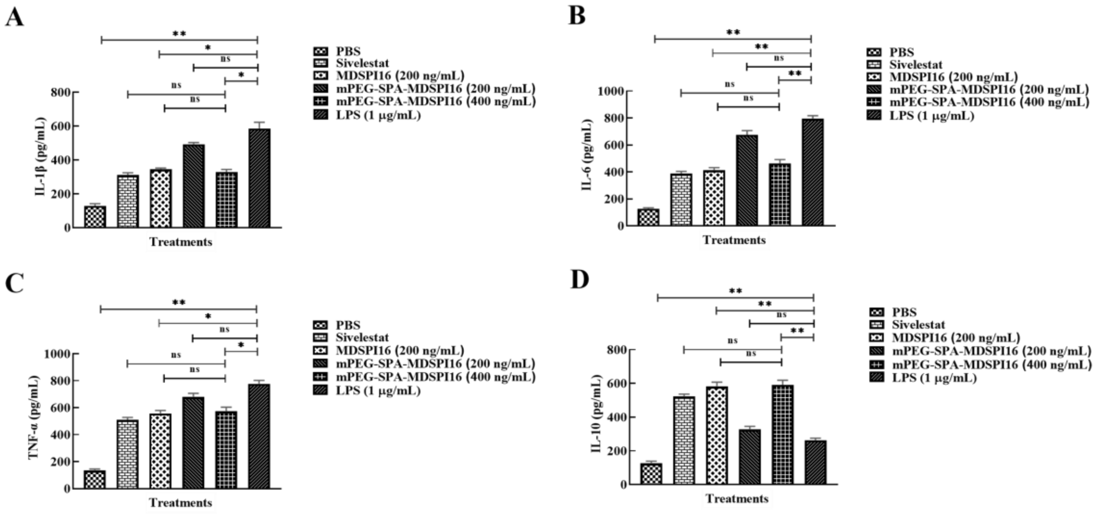

Figure S10. mPEG-SPA-MDSPI16 reduces the inflammatory cytokine levels secreted by LPS-induced neutrophils. The cells were induced by LPS, and then cultured at different concentrations of mPEG-SPA-MDSPI16 (200, 400ng/mL). The supernatant was collected and detected by ELISA for(A) IL-1β, (B) IL-6, (C) TNF-*α* and (D) IL-10. Data represented as Mean ± SEM, n = 10/group. * $p < 0.05$, ** $p < 0.01$, ns, $p > 0.05$ vs. the LPS-treated group; ns, $p > 0.05$ vs. the mPEG-SPA-MDSPI16(400 ng/mL) treatment group. P-values were determined using an unpaired two-tailed Student' s t-test.

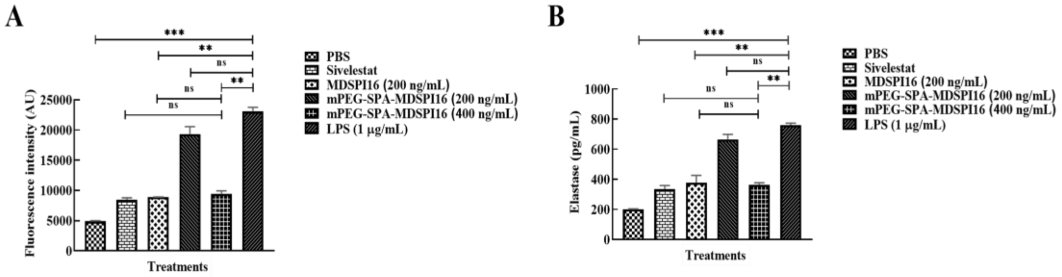

Figure S11. The produced level of ROS and neutrophil elastase by LPS-induced neutrophils. (A)The level of ROS produced by LPS-induced neutrophils. (B) Effect of mPEG-SPA-MDSPI16 on expression of neutrophil elastase in mice. Data represented as Mean ± SEM, n = 10/group. ** $p < 0.01$, *** $p < 0.001$, ns, $p > 0.05$ vs. the LPS-treated group; ns, $p > 0.05$ vs. the mPEG-SPA-MDSPI16(400 ng/mL) treatment group. P-values were determined using an unpaired two-tailed Student' s t-test.

Supplementary Table

Table S1 Pharmacokinetic parameters of MDSPI16 after subcutaneous administration in rats

Parameter	Quantitative value	Unit
$AUC_{0\text{-}t}$	86564	ng•mL-1 *h
$AUC_{0\text{-}\infty}$	93738	ng•mL-1 *h
$MRT_{0\text{-}t}$	3.62	h
$T_{1/2\beta}$	1.79	h
CL	4.44	min/(mL*kg)
V	0.688	L/kg

Table S2 Pharmacokinetic parameters of MDSPI16 after intramuscular administration in rats

Parameter	Quantitative value	Unit
AUC0-t	94380	ng•mL-1 *h
AUC0-∞	129115	ng•mL-1 *h
MRT0-t	6.12	h
T1/2β	4.08	h
CL	3.23	min/(mL*kg)
V	1.14	L/kg

Table S3 Pharmacokinetic parameters of MDSPI16 and mPEG-SPA-MDSPI16 after intravenous administration in rats

Parameter	MDSPI16	mPEG-SPA-MDSPI16	Unit
AUC0-t	160233	945242.45	ng•mL-1 *h
AUC0-∞	169861	1615026.5	ng•mL-1 *h
MRT0-t	1.57	2.84	h
T1/2β	1.79	7.17	h
CL	4.44	12	min/(mL*kg)
V	0.231	0.128	ng/mL

Table S4 Real-Time PCR primer sequences

Gene	Primer sequence (5′–3′)
IL-10	F：5'-GCCAGAGCCACATGCTCCTA-3'
	R：5'-GATAAGGCTTGGCAACCCAAGTAA-3'
IL-6	F：5'-CCACTTCACAAGTCGGAGGCTTA-3'
	R：5'- CCAGTTTGGTAGCATCCATCATTTC-3'
iNOS	F：5'-TGCCACGGACGAGACGGATAG-3'
	R：5'-CTCTTCAAGCACCTCCAGGAACG-3'
COX-2	F：5'-ATTCCAAACCAGCAGACTCATA-3'
	R：5'-CTTGAGTTTGAAGTGGTAACCG-3'
IL-1β	F：5'-TCCAGGATGAGGACATGAGCAC-3'
	R：5'-GAACGTCACACACCAGCAGGTTA-3'
TNF-α	F：5'-CCTATGTCTCAGCCTCTTCTCAT-3'
	R：5'-CACTTGGTGGTTTGCTACGA-3'
β-actin	F：5'-AAATGGTGAAGGTCGGTGTGAAC-3'
	R：5'- CAACAATCTCCACTTTGCCACTG-3'
